# Trade‐offs between morphology and thermal niches mediate adaptation in response to competing selective pressures

**DOI:** 10.1002/ece3.5990

**Published:** 2020-01-10

**Authors:** Stella F. Uiterwaal, Ian T. Lagerstrom, Thomas M. Luhring, Miranda E. Salsbery, John P. DeLong

**Affiliations:** ^1^ School of Biological Sciences University of Nebraska ‐ Lincoln Lincoln NE USA

**Keywords:** climate change, evolution, morphology, Paramecium, predation, trade‐off

## Abstract

The effects of climate change—such as increased temperature variability and novel predators—rarely happen in isolation, but it is unclear how organisms cope with multiple stressors simultaneously. To explore this, we grew replicate *Paramecium caudatum* populations in either constant or variable temperatures and exposed half to predation. We then fit thermal performance curves (TPCs) of intrinsic growth rate (*r*_max_) for each replicate population (*N* = 12) across seven temperatures (10°C–38°C). TPCs of *P. caudatum* exposed to both temperature variability and predation responded only to one or the other (but not both), resulting in unpredictable outcomes. These changes in TPCs were accompanied by changes in cell morphology. Although cell volume was conserved across treatments, cells became narrower in response to temperature variability and rounder in response to predation. Our findings suggest that predation and temperature variability produce conflicting pressures on both thermal performance and cell morphology. Lastly, we found a strong correlation between changes in cell morphology and TPC parameters in response to predation, suggesting that responses to opposing selective pressures could be constrained by trade‐offs. Our results shed new light on how environmental and ecological pressures interact to elicit changes in characteristics at both the individual and population levels. We further suggest that morphological responses to interactive environmental forces may modulate population‐level responses, making prediction of long‐term responses to environmental change challenging.

## Graphical abstract

To explore how well organisms can cope with multiple simultaneous selective pressures—such as those produced by climate change—we exposed *Paramecium caudatum* populations to both temperature variability and predation. Our findings suggest that these two stressors produce conflicting pressures on both thermal performance and cell morphology, making prediction of long‐term responses to environmental change challenging. We further found a strong correlation between changes in cell morphology and thermal performance curve parameters in response to predation, suggesting that responses to opposing selective pressures could be constrained by trade‐offs.

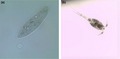

## 1 INTRODUCTION

Organisms often face multiple challenges simultaneously, including predation, resource availability, disease, and climate. Although adaptation to individual stressors is well studied, it is unclear how well organisms can adapt concurrently to multiple selective pressures (Condon, Cooper, Yeaman, & Angilletta, 2014; Frazier, Huey, & Berrigan, 2006; Sinclair et al., 2016). The effectiveness of adaptations may be reduced or constrained when responding to multiple stressors (Luhring, Vavra, Cressler, & DeLong, 2019).

Organisms may experience simultaneous selective pressures as a result of climate change. Local climates may experience, for example, changes in the mean and variance of temperature along with changes in rainfall (IPCC, 2019). These altered climate patterns further facilitate changes to biotic components such as community structure and disease prevalence. In Hawaii, for instance, climate change coupled with introduced diseases such as avian malaria has been implicated in the loss of native birds (Atkinson & LaPointe, 2009). In the eastern Mediterranean Sea, warming water has been accompanied by invasions of tropical species via the Suez canal (Azzurro, Maynou, Belmaker, Golani, & Crooks, 2016; Rijn, Buba, DeLong, Kiflawi, & Belmaker, 2017). And across continents, pollution and fishing threaten penguin populations already stressed by climate change (Trathan et al., 2015). As organisms increasingly face concurrent selective pressures, it is paramount to understand whether populations can adapt to such novel challenges.

Thermal performance curves (TPCs) characterize organism‐ or population‐level performance as functions of temperature, and they often are used to study how organisms respond to selective pressures (Angilletta, 2009; Huey & Kingsolver, 1989; Sinclair et al., 2016). TPCs are typically unimodal, exhibiting a shallow rise toward a peak followed by a decline at hotter temperatures. TPCs indicate thermal niche by reflecting underlying traits that may be phenotypically plastic or responsive to selection pressures, such as temperature (Angilletta, 2009; DeLong et al., 2018; Huey & Kingsolver, 1989; Krenek, Petzoldt, & Berendonk, 2012; Vasseur et al., 2014). The width and height of a TPC and the location of the optimal temperature are therefore expected to at least somewhat reflect a population's local climate (DeLong et al., 2018), allowing TPCs to be used as a lens to predict the consequences of climate change (Deutsch et al., 2008; Krenek et al., 2012). TPCs might also be expected to adjust as climate changes. Long‐term acclimatization, for example, at constant moderate temperatures or in variable climates can produce broader thermal tolerance (Bozinovic et al., 2011; Condon et al., 2014; Luhring & DeLong, 2017; Sunday, Bates, & Dulvy, 2011), although other work suggests that decreased thermal variation can encourage specialism (Gilchrist, 1995). TPCs also may shift to reflect exposure to warmer or colder thermal regimes (Alexander & McMahon, 2004; Luhring & DeLong, 2017; Padfield, Yvon‐Durocher, Buckling, Jennings, & Yvon‐Durocher, 2016).

Because species interactions are temperature‐dependent (Englund, Öhlund, Hein, & Diehl, 2011; Uiterwaal & DeLong, 2018), predation and parasitism also can change the shape of TPCs (Grigaltchik, Ward, & Seebacher, 2012; Grigaltchik, Webb, & Seebacher, 2016). For example, populations of *Paramecium aurelia* exposed to predation show a more rapid increase in growth as temperature increases and a more rapid decline as temperatures decrease than populations not exposed to predation (Luhring & DeLong, 2016). Similarly, *Daphnia magna* show changes in body size, population growth rate, and life‐history traits in response to predation risk (Luhring, Vavra, Cressler, & DeLong, 2018; Luhring et al., 2019; Tseng, Bernhardt, & Chila, 2019), and bacteriophage presence alters TPCs in the bacterium *Pseudomonas fluorescens* (Padfield, Castledine, & Buckling, 2019).

Changes in TPCs reflect underlying changes in life‐history traits such as fecundity or survivorship (Stearns, 1992). Many traits are simultaneously responsive to temperatures and species interactions (Luhring et al., 2018; Padfield et al., 2019; Salsbery & DeLong, 2018). Thus, organisms in natural environments exposed to climate shifts may face multiple selective forces on the same traits that could facilitate or counteract adaptation to changes in temperature. Although the separate effects of ecological and environmental factors on thermal performance have been studied widely, it is increasingly clear that we must also understand how predation and climate interact to affect TPCs across temperatures (Bernhardt, Sunday, Thompson, & O’Connor, 2018; Grigaltchik et al., 2012; Sinclair et al., 2016; Zarnetske, Skelly, & Urban, 2012).

**Figure 1:**
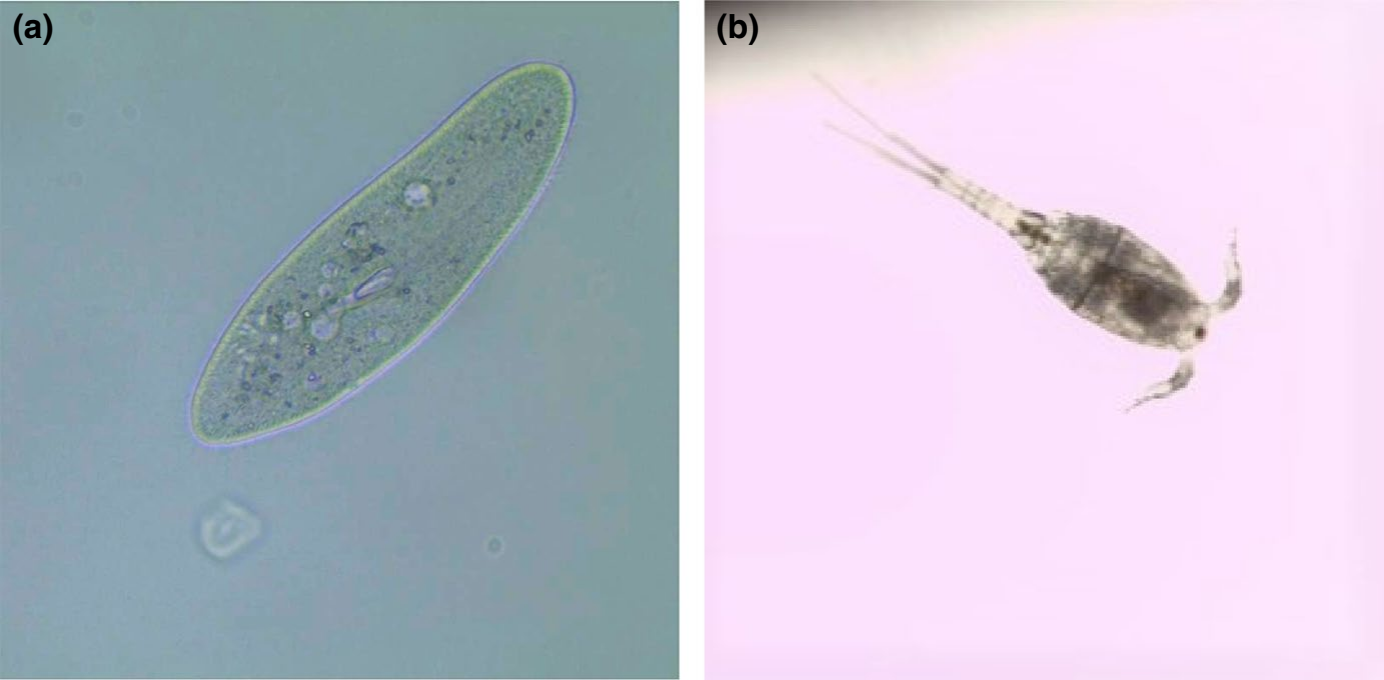
Study organisms. (a) *Paramecium caudatum* (b) *Eucyclops agilis*

To understand the interactive effects of temperature variability and predation on the shape of TPCs, we fit population growth rate TPCs (hereafter *r*TPCs) of *Paramecium caudatum* (Figure 1a) populations with differing predation and temperature variation exposure histories. For the predator treatments, we used copepods (Figure 1b), a ubiquitous aquatic predator that forages heavily on protists (Kalinoski & DeLong, 2016). For temperature variability, we exposed *P. caudatum* to either a constant temperature (29 ± 0°C) or regular temperature fluctuations (29 ± 4°C). Because both predation and temperature may cause differences not just in characteristics of population growth but also in traits of individuals (Atkinson, 1994; Laurila, Crochet, & Merilä, 2001; Luhring & DeLong, 2017; Tollrian, 1995), we looked for changes in cell morphology as well. We predicted that predation would increase the height of *r*TPCs (a faster life history) and that temperature variation would broaden the *r*TPCs (shifting toward thermal generalism) (Huey & Kingsolver, 1989). We also predicted that changes in cell size or shape would accompany shifts in population growth, since division at a smaller cell volume is one way to achieve faster population growth, and cell shape is linked to predation risk in *Paramecium* (Hammill, Petchey, & Anholt, 2010). Our results agree with some predictions but unexpectedly show considerable variation in the response of *r*TPCs to both temperature variation and predation. Upon closer investigation, we found that the morphological response to predation was strongly related to the TPC response to predation, suggesting trade‐offs that constrain the overall response to competing selective forces.

## 2 MATERIALS & METHODS

### 2.1 Study organisms

We collected *P. caudatum* and *Eucyclops agilis* copepods from two ponds in Lancaster County, Nebraska: one at Spring Creek Prairie Audubon Center and one on the University of Nebraska—Lincoln East Campus. Organisms were collected in August 2017, when daily temperatures were between 15°C and 30°C (NOAA, 2019). *Paramecium caudatum* from both sources was mixed together thoroughly and divided evenly into six 10 cm Petri dishes to produce starting populations of approximately 50 cells per dish. We maintained *P. caudatum* in two dark growth chambers at either 29 ± 0°C or 29 ± 4°C (a 12‐hr smooth cycle between 25°C and 33°C). Each growth chamber contained three of the Petri dishes. We kept the *P. caudatum* populations in their respective climates for 5 months (~300 generations) prior to initiating our predation treatment. We maintained *P. caudatum* and copepods separately in bacterized media, which consisted of nine parts filtered and autoclaved pond water (ACPW) (a mixture of water from both source ponds) and one part liquid protozoa medium from Carolina Biological Supply (Burlington, NC, USA) inoculated with bacteria isolated from the source ponds. Each Petri dish contained one autoclaved rice grain as a carbon source. To encourage exponential growth of *P. caudatum* and to maintain high bacterial and nutrient levels, we discarded half of each *Paramecium* culture three times a week and replaced the lost volume with fresh bacterized media. We kept copepods at room temperature (~23°C) supplied with a surplus of *P. caudatum*, adding bacterized media as necessary to offset evaporative loss.

### 2.2 Predation exposure

We separated each of the six populations into two replicates, designated as predation and no‐predation treatments, resulting in 12 experimental replicates (2 temperature treatments × 3 populations per temperature treatment × 2 predation treatments). We pipetted two copepods, washed twice in ACPW, and starved for 24 hr at either 29 ± 0°C or 29 ± 4°C, along with 50 µl of ACPW into each predation dish. We also added 50 µl of ACPW to each no‐predation dish to minimize variation between the predation and no‐predation treatments’ microbial communities. After 24 hr, we removed the predators. To avoid any effects of differing population densities due to predation between predation and no‐predation treatments, we collected 100 cells from each dish and added these to a fresh 20 ml mixture of one part bacterized media and one part sterile media (bacteria‐free ACPW and protozoa medium) in a clean dish. Two days later, we added an additional 10 ml of bacterized media to each dish. We repeated this process once a week for 4 weeks (~56 generations).

### 2.3 Thermal performance curves

Due to the logistic difficulty of estimating 12 *r*TPCs simultaneously, we conducted population growth tests on three consecutive days, testing four of 12 experimental replicates each day. We randomly assigned the replicates to a specific day, but we always tested the predation and no‐predation treatments for a given local climate and population combination on the same day. We conducted six trials per replicate in growth chambers at seven temperatures (10, 20, 25, 29, 33, 36, and 38°C). Thus, the day before a trial, we prepared 168 35‐mm petri dishes (4 replicates × 6 trials × 7 temperatures). For each trial, we added seven *P. caudatum* from the appropriate treatment in 100 µl of media to each dish. In laboratory pilots, we found that initiating dishes with seven cells minimized both stochastic loss of cells and density dependence. Then, we added 1.5 ml of bacterized media (a surplus of food) to each dish and placed them in temperature‐controlled growth chambers overnight (without an acclimation period). The next day, we counted the number of *P. caudatum* in each dish and recorded the total time each dish spent in the growth chamber. By starting with a small population, providing an abundance of bacterial food, and leaving dishes for a relatively short period of time, we intended to promote exponential growth, enabling us to use overnight growth rates as an approximation for maximum growth rates.

### 2.4 Cell morphology

We obtained measurements of *P. caudatum* length (to the nearest 0.01 μm), width (to the 0.01 μm), and volume (to the 100 μm^3^) from each replicate during the same week as the *r*TPC experiments. We took cell samples from the incubators at random during the week and measured all cells at room temperature (~23°C) using a FlowCam (Fluid Imaging Technologies) fitted with a 4× objective lens. The mean number of observations was 422 cells per experimental replicate (Table S1). We only used images depicting entire cells for size measurements; images showing only parts of cells were discarded.

### 2.5 Data analysis

We calculated maximum population growth rate (*r*_max_) for each replicate using the standard model for exponential growth: $r=\frac{\ln N_{t}\;/N_{0}\;N_{0}}{t}$, where *N_t_* is the final population size, *N_0_* is the initial population size in a replicate dish (7), *r* is the population growth rate, and *t* is the total time (time from placement in growth chamber to counting of cells). Because *r* cannot be calculated when the final population size is zero, we calculated *r* using a final population size of 1 when no cells survived.

To assess the temperature dependence of *r*_max_ for each of the 12 replicates, we generated 1,000 datasets using stratified bootstraps with replacement and fit them to the Lactin‐2 function (Lactin, Holliday, Johnson, & Craigen, 1995):

(1) $$r_{\max}=e^{\rho x}-e^{\rho T_{\max}-\frac{T_{\max}-x}{\Delta T}}+\lambda$$

Although the parameters *ρ*, *T_max_*, *∆T*, and *λ* do not have clear biological interpretations, this function describes well the unimodal shape of *r*TPCs and thus provides a good characterization of the overall *r*TPC shape. Unlike many available functions, the Lactin‐2 function also allows negative rates, allowing for clear identification of the upper and lower critical temperatures (DeLong et al., 2017). We used the median values and 95% quantiles of the resulting distributions of Lactin‐2 parameters and *T*_opt_, *r*_opt_ (*r*_max_ at *T*_opt_), *CT*_min_, and *CT*_max_ of each bootstrapped dataset as estimates and confidence intervals, respectively. *CT*_min_ and *CT*_max_ are the lower and upper critical temperatures, respectively, beyond which population growth rate becomes negative and the population cannot persist. We obtained these critical temperatures by finding the roots of parameterized (Equation 1) for each population. *T*_opt_ is the temperature at which the population growth rate was highest (*r*_opt_). For each matched set of Predator–No Predator treatments, we calculated the difference between 1,000 randomly selected values from the *T*_opt_ distribution of both treatments. We then calculated 95% quantiles of the resulting distribution to determine whether the difference between *T*_opt_ values was nonzero. We repeated this process for *r*_opt_, *CT*_min_, and *CT*_max_, and calculated thermal breadth as the difference between *CT*_min_ and *CT*_max_.

Using size measurements from the FlowCam, we calculated cell volume for each cell assuming *P. caudatum* cells were shaped as prolate spheroids:

$V=\frac{4}{3}\pi LW^{2},$ where *V* is cell volume (μm^3^), *L* is half of the cell length (μm), and *W* is half of the cell width (μm). We calculated cell shape as the ratio of length over width $\left(\frac{L}{W}\right)$. A large ratio indicates long, narrow cells while a ratio of one indicates a round cell. We then analyzed cell length, width, volume, and shape with linear mixed‐effect models using temperature variability, predator treatment, and an interaction between temperature and predation as predictor variables. We used population as a random effect to account for the hierarchical structure of our data (Gibert, Allen, Hruska, Ron, & DeLong, 2017).

Finally, we looked for correlations between changes in *r*TPC parameters and cell morphology in response to predation. We calculated the differences in mean morphological traits (cell length, shape, and volume) and *r*TPC parameters between all six pairs of replicates exposed to either predators or no‐predator treatments. We then used Pearson's correlation to determine whether across populations change in morphology was linked to change in thermal niche. We used Matlab for all analyses.

## 3 RESULTS

**Figure 2:**
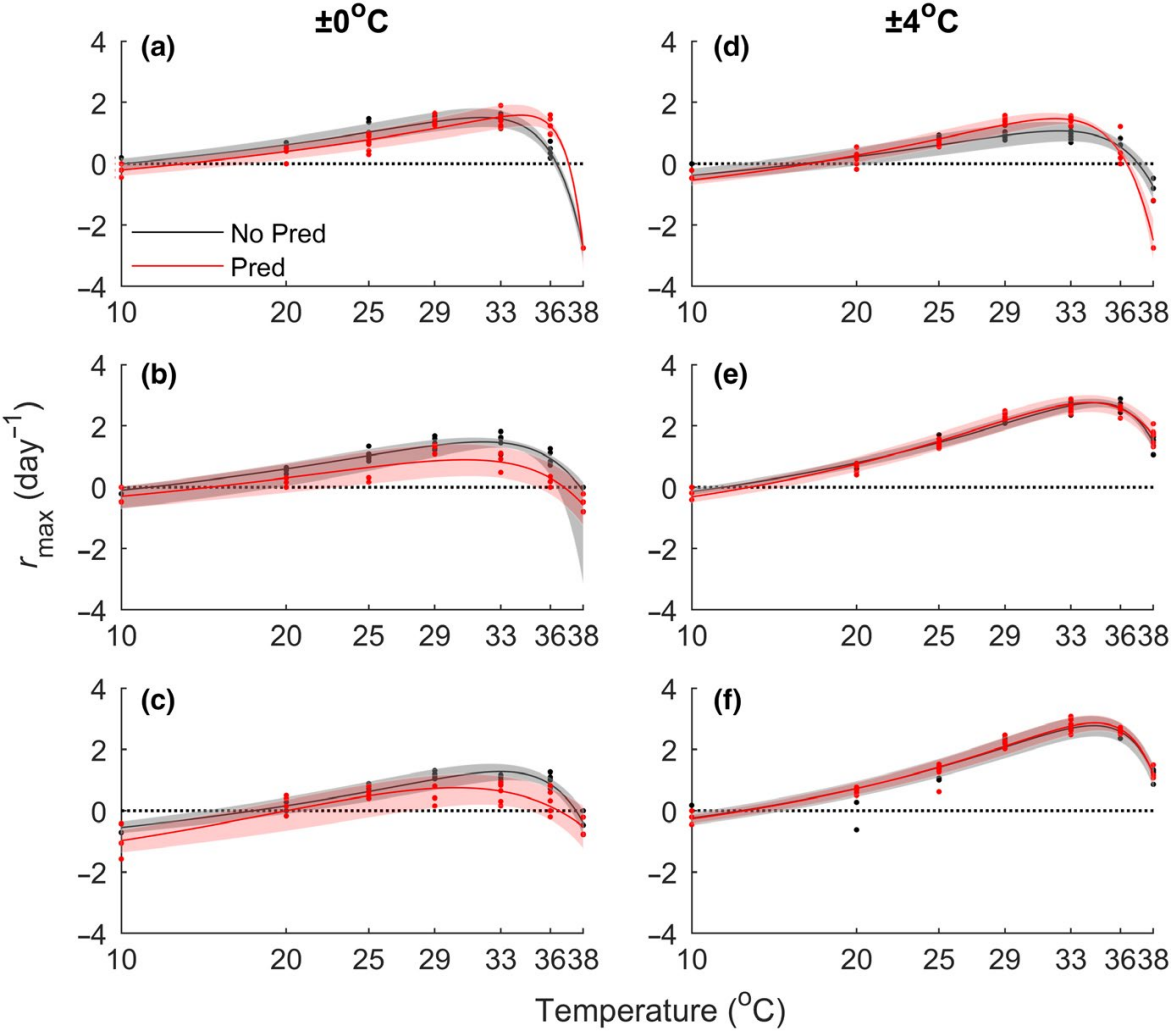
*Paramecium caudatum* TPCs for instantaneous growth rate (*r*_max_). Dots represent data points from the *r*TPC experiment. Bootstrapped curves were fitted to a Lactin‐2 function. Shaded regions show the 95% confidence interval for each curve. (a–c) Populations grown at a constant temperature (29 ± 0°C). (d–f) Populations grown at a variable temperature (29 ± 4°C). Note that matched pairs of replicates are shown in the same panel; alignment of panels (a–c) next to panels (d–f) is arbitrary. TPCs, thermal performance curves

**Figure 3:**
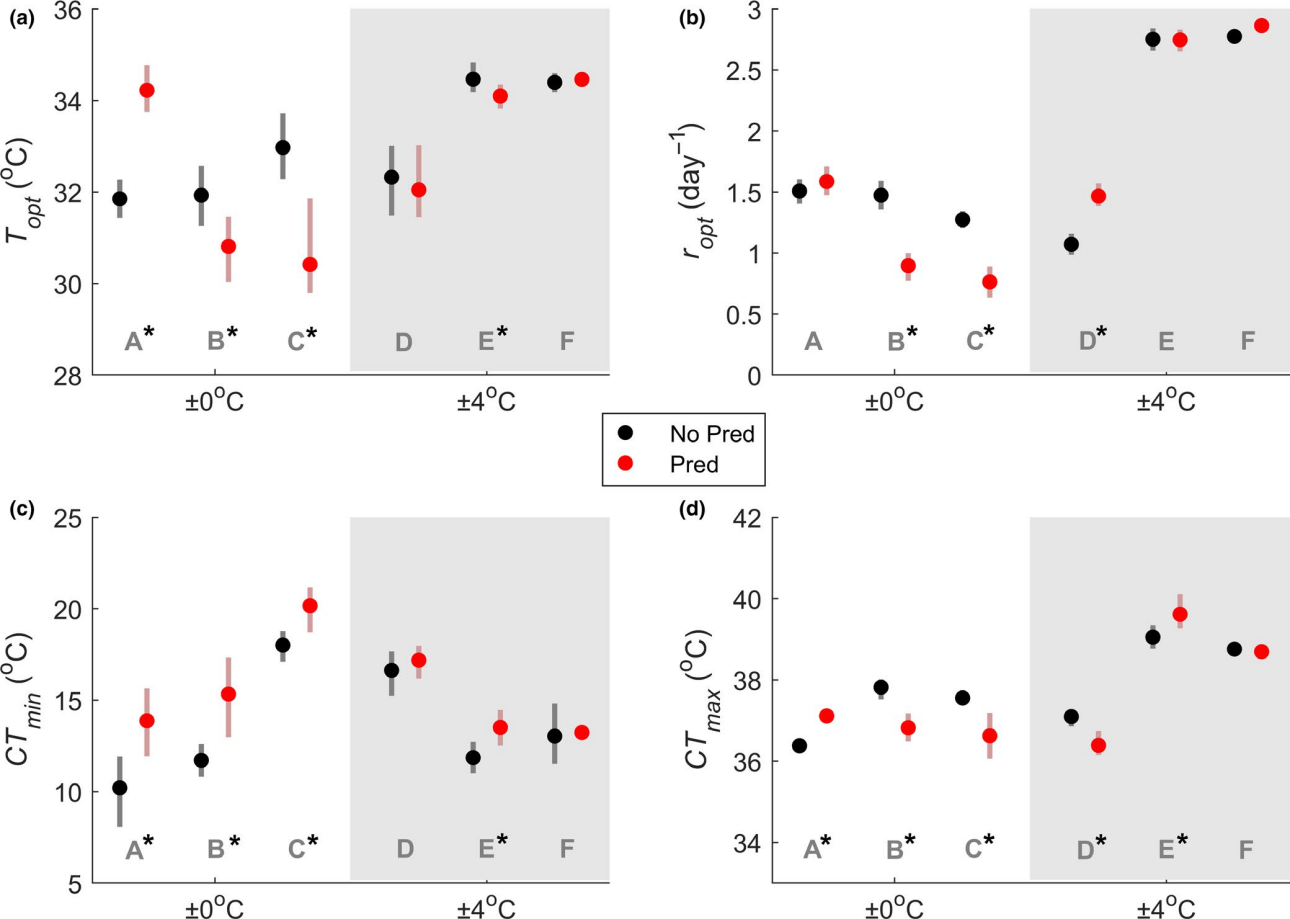
Shape parameters from *Paramecium caudatum r*TPCs for each treatment. (a) *T*_opt_, (b) *r*_opt_, (c) *CT*_min_, and (d) *CT*_max_. Gray letters “A” through “F” correspond to panels in Figure 1. An asterisk denotes a significant difference between predation and no‐predation treatments

Responses of *P. caudatum* TPCs to predation and variation in temperature were variable and not consistent with our predictions (Figure 2). In general, predation tended to lower *r*TPCs of *P. caudatum* grown at constant temperatures (29 ± 0°C) but not those grown in variable temperatures (29 ± 4°C) (Figures 2 and 3). At a constant temperature (replicates A, B, and C), predation always caused a change in *T*_opt_, *CT*_min_, and *CT*_max_. In two of three replicates (B and C), predation also affected *r*_opt_. The directions of these changes were largely unpredictable. In all replicates at a constant temperature (A, B, and C), predation decreased cold tolerance (warmer *CT*_min_). This was accompanied by decreased heat tolerance (colder *CT*_max_) in two replicates (B and C), indicating that predation narrowed the thermal range in which populations can persist (Table 1). One predator replicate (A) grown at a constant temperature had both warmer lower and upper critical temperatures, pushing the TPC to the right. This same replicate showed a reverse effect of predation on *T*_opt_ compared to the other ±0°C replicates (warmer instead of cooler) and was the only 29 ± 0°C replicate which showed no effect of predation on *r*_opt_. The other two replicates grown at 29 ± 0°C (B and C) showed a decrease in *r*_opt_ with predation.

**Table 1:** Thermal breadth of instantaneous growth rate (*r*_max_) TPCs of *Paramecium caudatum* in different predation and temperature variability treatments

Pair	Temperature variability	Thermal breadth (℃)		
		No predation	Predation	ΔBreadth
A	N	26.17	23.24	−2.94
B	N	26.11	21.49	−4.62
C	N	19.54	16.46	−3.08
D	Y	20.46	19.20	−1.26
E	Y	27.19	26.10	−1.09
F	Y	25.72	25.46	−0.26

Note

Pairs of populations “A” through “F” correspond to panels in Figure 2.

Abbreviation: TPCs, thermal performance curves.

TPCs of populations grown in variable temperatures (reps. D, E, and F) tended to be less responsive to predation, showing almost identical curves between predator and nonpredator treatments in two replicates (E and F) (Figures 2 and 3). At 29 ± 4°C, predation affected the optimum temperature in only one replicate (E). *r*_opt_ was unaffected by predation for two of the 29 ± 4°C replicates (E and F) and was increased by predation for one replicate (D). When predation did affect *r*_opt_, the direction of the effect was reversed between populations from constant and variable temperatures. Thus, our results indicate that although predation did not necessarily affect *r*_opt_ in either constant or variable temperatures, temperature variation and predation can interact to alter *r*_opt_. All predation replicates grown at 29 ± 0°C had a warmer lower critical temperature, but only one replicate at 29 ± 4°C showed the same effect (E). The lower critical temperature of the other two replicates (D and F) was unaffected by predation. This suggests that predation tends to reduce cold tolerance, although this is less likely in variable climates. The effect of predation on the upper critical temperature varied widely for populations raised in variable temperatures, causing either an increase (rep. E), decrease (rep. D), or no change (rep. F) in *CT*_max_. Predation also narrowed the thermal breadth when temperature varied, but this effect was reduced compared to the constant temperature treatments (Table 1). For each replicate, parameter estimates for bootstrapped Lactin‐2 curves are given in Table S2.

In contrast, temperature variation and predation had clear impacts on cell morphology (Table 2). Combined variable temperatures and predation caused a decrease in cell length, which was not seen when temperature variability and predation acted on *P. caudatum* populations independently. Independent effects of temperature variability and predation became apparent when looking at both length and width (cell shape). More variation in temperature caused *P. caudatum* to be rounder (decreased length:width ratio), while predation caused cells to be more torpedo‐shaped (increased length:width ratio). The interaction between temperature variability and predation caused cells to have an intermediate shape compared to *P. caudatum* affected independently by either temperature variability or predation. Despite these differences in cell shape, cell volume was conserved across treatments.

**Table 2:** Linear mixed‐effects model results for *Paramecium caudatum* cell size measurements for each treatment

Term	Estimate	SE	*p*‐value
Length			
**Intercept**	**152.560**	**10.278**	**<.001**
Temperature (±4)	4.071	14.538	.779
Predator	5.294	3.502	.131
**Temperature (±4):Predator**	**−10.507**	**4.860**	**.031**
Width			
**Intercept**	**33.060**	**1.710**	**<.001**
Temperature (±4)	4.179	2.420	.084
Predator	−0.015	1.552	.992
Temperature (±4):Predator	−0.051	2.185	.981
Shape (Length:Width)			
**Intercept**	**4.755**	**0.090**	**<.001**
**Temperature (±4)**	**−0.433**	**0.128**	**<.001**
**Predator**	**0.273**	**0.136**	**.045**
**Temperature (±4):Predator**	**−0.384**	**0.188**	**.041**
Volume			
**Intercept**	**90,169**	**16,147**	**<.001**
Temperature (±4)	32,274	22,839	.158
Predator	754	10,504	.943
Temperature (±4):Predator	−5816	14,786	.694

Note

Significant terms are bolded.

**Figure 4:**
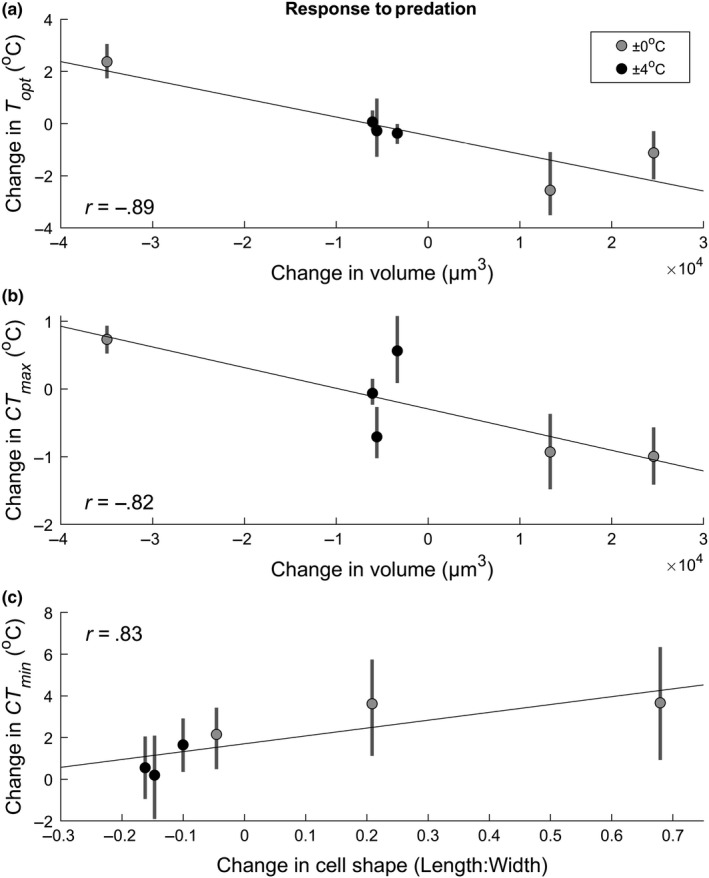
Correlations between changes in morphological and thermal traits. (a) *T*_opt_ versus cell volume, (b) *T*_opt_ versus cell volume, and (c) *CT*_min_ versus cell shape. Points represent differences in traits between the six pairs of populations subjected to predation or no‐predation treatments. Bars show 95% confidence intervals

Lastly, we found that *P. caudatum* that became larger in response to predation also shifted their *r*TPCs cooler, as there was a strong negative relationship between cell volume and both the *T*_opt_ and *CT*_max_ (*T*_opt_: *r* = −0.89, *p* = .017, *CT*_max_: *r* = −0.82, *p* = .048) (Figure 4). We also found a strong positive relationship between cell shape and *CT*_min_ (*r* = 0.83, *p* = .039), indicating that populations that became more torpedo‐shaped in response to predation also became less cold tolerant under predation. Other correlations between changes in morphological and thermal traits in response to predation were not significant (Figure S1).

## 4 DISCUSSION

Climate projections suggest that there will be increases in both the mean and variance in environmental temperature (IPCC, 2019), and previous work suggests that *Paramecium* is vulnerable to these changes (Krenek et al., 2012). Given that *r*TPCs presumably reflect an organism's locally adapted climate, our *Paramecium* cultures exposed to increased temperature variance might have been expected to show wider and shallower *r*TPCs than *Paramecium* exposed to a constant temperature, consistent with a thermal specialist‐generalist trade‐off (Duncan, Fellous, Quillery, & Kaltz, 2011; Huey & Kingsolver, 1989). Although ± 4°C populations showed some indication of higher growth at hotter temperatures, this did not come at the cost of lower population growth overall (Figures 2 and 3). Furthermore, the overall shifts with temperature variance appeared somewhat unpredictable, with variable temperature *r*TPCs both higher and lower than constant temperature *r*TPCs. Likewise, cells exposed to predation showed an inconsistent response in the *r*TPC (i.e., right shift, left shift, no shift, and down shift; Figure 2), indicating that increased risk of predation did not generally lead to faster divisions. Together, our results suggest that the directional shifts in thermal niches that might be expected given single selective forces might not occur when multiple stressors are simultaneously present.

In contrast, both temperature variation and predation risk had significant and consistent effects on *Paramecium* cell shape (Table 2). Temperature variation led to rounder cells while predation led to longer, narrower cells. These effects thus ran counter to each other, and the effect of temperature variation on cell shape was reduced by the presence of predators (significant variation × predation interaction). This could be because cell volume in protists is highly plastic, facilitating consistent responses to stressors and not reliant on the occurrence of new mutations or high standing genetic variation. Morphological changes have been shown to reduce predation in another *Paramecium* species, although interestingly those cells become wider—rather than narrower—perhaps because the predator was gape‐limited (Hammill et al., 2010). Although the benefits of a narrower cell shape are unclear in our case, predation may have encouraged longer, thinner cells that increased maneuverability, or swimming speeds, allowing cells to avoid predators more successfully, whereas rounder cells may help control the exchange of gases and resources across membranes in changing temperatures (Okie, 2013).

Surprisingly, even though changes in cell volume can facilitate changes in division time, there were no uniform effects of our treatments on cell volume. These results indicate that the functional consequences of cell shape, such as gas‐exchange, movement ability, or foraging strategy, may be key to maintaining fitness given both abiotic and biotic stressors. Furthermore, this suggests that changes in biomass do not compensate for the observed changes in population growth rates (*r*TPCs) (Padfield, Buckling, Warfield, Lowe, & Yvon‐Durocher, 2018), validating the choice of population size rather than biomass as our metric of growth.

Despite having just three replicate populations for each treatment, the variation within the three replicates suggests that a predictable pattern of evolution in response to two opposing stressors is unlikely, especially since there are multiple traits under selection. However, our results further suggest that apparently haphazard changes in the *r*TPCs of *Paramecium* are linked to a possible trade‐off between morphological and thermal traits (Figure 4). Although there was no consistent effect of either temperature variation or predation on cell volume, there were predictable changes that occurred between paired no‐predation/predation replicates in different temperature variation treatments. In the populations where predation led to smaller cell volume, both *T*_opt_ and the *CT*_max_ increased, while in the populations where predation led to larger cell volume, *T*_opt_ and *CT*_max_ decreased. Dispersion of replicates along this possible trade‐off line further suggests that temperature variability constrained the response of *Paramecium* to predation, as these populations showed similar traits clustered around the origin (Figure 4a,b). In contrast, populations in constant temperatures showed either a strong positive or negative shift in cell size, accompanied by a strong shift in *r*TPC. Variation across replicates also occurred with cell shape, accompanied by changes in the *CT*_min_ (Figure 4c). Cells that became rounder showed less change in *r*TPCs, and this outcome was typical for the populations in a variable environment. Populations in the constant environment, on the other hand, showed varying degrees of becoming longer and narrower, accompanied by an increasingly strong rightward shift in the lower critical temperature. The relationship between morphological response to predation and the *r*TPC response to predation suggests that trade‐offs constrain the overall response to competing selective forces. Furthermore, our results suggest that the presence of one stressor can determine the ability to respond to a second stressor. While populations from the ±4°C treatments were clustered around the origin in each paired set of morphological and thermal traits (indicating an inability to respond to the second stressor—predation), populations exposed to a constant temperature appeared free to move in either direction along the trade‐off.

Our results highlight the challenges organisms may experience when faced with more than one stressor at a time. When multiple stressors produce selective pressure in opposite directions, it may be difficult or impossible to simultaneously adapt to both pressures, resulting in adaptation to just one of the stressors or partial adaptation (Table 2). In this case, when faced with thermally variable environments, populations may be limited in their ability to respond to predation at all (Figure 4). Finally, connections between different traits (possible genetic correlations) might constrain adaptation to shifts in pairs of traits rather than independent trait evolution. This latter process may underlie some of the apparent haphazard responses of *Paramecium* to temperature variability and predation risk.

How our populations started down a path toward right‐shifted *r*TPCs and large cells or the reverse is unclear, but it is possible that chance mutations led toward different potential solutions to the risk of predation. Once a small adaptation in one direction has occurred, the population may be set on an evolutionary trajectory toward one particular solution rather than another. However, because we did not observe mating cells during our experiment, the responses to treatments may not be due to new mutations or recombinations. Instead, the differences may have arisen from standing genetic variation, phenotypic plasticity, or epigenetics.

Nonetheless, our results point to substantial challenges in predicting the response of organisms to changing climates in natural systems with multiple stressors. These findings show that, while predation and temperature are often considered separately, organisms may respond in dramatically different ways when both challenges are considered in tandem. Such tandem stressors are increasingly likely, as not only are climates changing, but predator invasions are occurring rapidly in many parts of the world (Doherty, Glen, Nimmo, Ritchie, & Dickman, 2016), even as top predators are being reduced (Ripple et al., 2014), generating no‐analogue predator‐prey interactions that might undermine the ability of these populations to adapt to climate change. Although the predators in our study were not adapted to a variable climate, we might expect their predation to become more efficient as they did so, increasing selective pressure due to predation. In general, adaptation can become increasingly complex as multiple species each respond to thermal challenges (West & Post, 2016). Temperature variation alone has profound effects (Frazier et al., 2006), but accounting for species interactions may provide more realistic predictions of the potential paths of adaptation (Zarnetske et al., 2012). Our results clarify that responses to environmental change—especially in the face of opposing selective forces—might be highly unpredictable, especially without additional understanding of the interactive effects of multiple stressors and the links among traits that may constrain adaptive options.

## AUTHOR CONTRIBUTIONS

SFU, TML, and JPD conceived the experiment. SFU, ITL, and MES conducted the experiment. SFU and JPD analyzed the data. SFU and ITL wrote the first draft. All authors contributed to editing the manuscript.

## Data Availability

Data is available on the Dryad Digital Repsitory at ://doi.org/10.5061/dryad.pzgmsbcgb (Uiterwaal, Lagerstrom, Luhring, Salsbery, & DeLong, 2019).
