# Supplementary figures and images for: Trade‐offs between morphology and thermal niches mediate adaptation in response to competing selective pressures
Source: Ecol Evol. 2020 Jan 10;10(3):1368–77. doi: 10.1002/ece3.5990 (PMC7029080; doi:10.1002/ece3.5990)

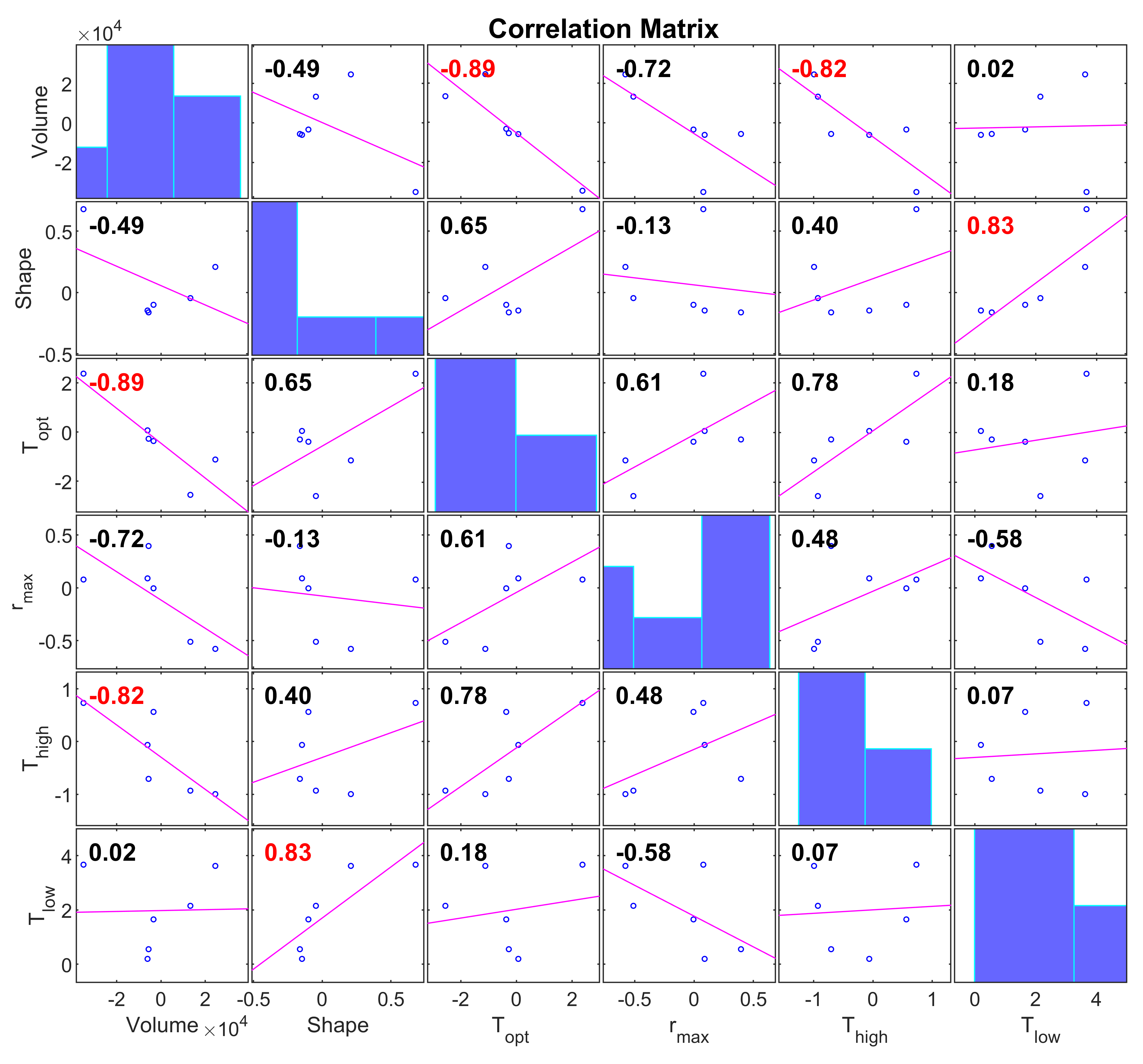

Supplement: Supplementary file 1 [file ECE3-10-1368-s001.tif]
