# Supplementary material for: Trade‐offs between morphology and thermal niches mediate adaptation in response to competing selective pressures
Source: Ecol Evol. 2020 Jan 10;10(3):1368–77. doi: 10.1002/ece3.5990 (PMC7029080; doi:10.1002/ece3.5990)
Supplement: Supplementary file 2 [file ECE3-10-1368-s002.docx]

**Appendix**

Figure A1. Correlation matrix for differences in morphology and *r*TPC parameters between pairs of populations exposed to predators and not exposed to predators. Numbers in subplots are Pearson’s correlation coefficients. Significant correlations are shown in red.

Table A1. Sample sizes for *Paramecium caudatum* cell morphology measurements. Pairs of populations “A” through “F” correspond to panels in Figure 2.

| **Temperature Variability** | **Pair** | **Predation** | **Sample size** |
| --- | --- | --- | --- |
| ±0℃ | A | N | 194 |
|  |  | Y | 446 |
|  | B | N | 341 |
|  |  | Y | 96 |
|  | C | N | 894 |
|  |  | Y | 212 |
| ±4℃ | D | N | 233 |
|  |  | Y | 922 |
|  | E | N | 675 |
|  |  | Y | 515 |
|  | F | N | 233 |
|  |  | Y | 307 |

Table A2. Parameter estimates and 95% confidence intervals for bootstrapped Lactin-2 curves of instantaneous growth rate (*r_max_*) TPCs of *Paramecium caudatum*. Pairs of populations “A” through “F” correspond to panels in Figure 2. Var = Temperature Variability. Pred = Predation. Y = Yes. N = No.

| Var | Pair | Pred | **Estimate** | | | |  | **Lower 95% CI** | | | |  | **Upper 95% CI** | | | |
| --- | --- | --- | --- | --- | --- | --- | --- | --- | --- | --- | --- | --- | --- | --- | --- | --- |
|  |  |  | ***∆T*** | ***λ*** | **ρ** | ***T_max_*** |  | ***∆T*** | ***λ*** | **ρ** | ***T_max_*** |  | ***∆T*** | ***λ*** | **ρ** | ***T_max_*** |
| ±0℃ | A | N | 1.95 | -1.45 | 0.04 | 37.41 |  | 1.72 | -1.59 | 0.03 | 37.33 |  | 2.20 | -1.32 | 0.04 | 37.48 |
|  |  | Y | 1.02 | -1.63 | 0.04 | 37.73 |  | 0.81 | -1.79 | 0.03 | 37.68 |  | 1.22 | -1.49 | 0.04 | 37.78 |
|  | B | N | 3.13 | -1.58 | 0.04 | 39.41 |  | 2.46 | -1.68 | 0.04 | 38.90 |  | 4.02 | -1.49 | 0.04 | 39.97 |
|  |  | Y | 3.74 | -1.73 | 0.04 | 39.49 |  | 2.97 | -2.05 | 0.03 | 38.91 |  | 5.14 | -1.51 | 0.04 | 40.34 |
|  | C | N | 2.37 | -2.02 | 0.04 | 39.20 |  | 1.74 | -2.17 | 0.04 | 38.78 |  | 3.18 | -1.88 | 0.04 | 39.76 |
|  |  | Y | 8.27 | -2.56 | 0.07 | 41.39 |  | 3.40 | -2.84 | 0.04 | 39.75 |  | 8.78 | -2.14 | 0.07 | 42.05 |
| ±4℃ | D | N | 2.45 | -1.81 | 0.04 | 38.86 |  | 1.89 | -1.96 | 0.03 | 38.53 |  | 3.25 | -1.64 | 0.04 | 39.29 |
|  |  | Y | 2.20 | -2.07 | 0.04 | 37.80 |  | 1.66 | -2.19 | 0.04 | 37.66 |  | 2.67 | -1.93 | 0.04 | 38.06 |
|  | E | N | 2.06 | -1.74 | 0.05 | 39.81 |  | 1.66 | -1.83 | 0.05 | 39.38 |  | 2.46 | -1.65 | 0.05 | 40.23 |
|  |  | Y | 2.94 | -1.97 | 0.05 | 40.69 |  | 2.50 | -2.12 | 0.05 | 40.23 |  | 3.55 | -1.84 | 0.05 | 41.38 |
|  | F | N | 1.94 | -1.85 | 0.05 | 39.50 |  | 1.76 | -2.06 | 0.05 | 39.31 |  | 2.17 | -1.70 | 0.05 | 39.69 |
|  |  | Y | 1.87 | -1.89 | 0.05 | 39.41 |  | 1.60 | -2.02 | 0.05 | 39.23 |  | 2.16 | -1.77 | 0.05 | 39.64 |
